# In-frame germline *TP53* variant impairs p53 oligomerization and predisposes to cancer

**DOI:** 10.1038/s41598-025-14684-8

**Published:** 2025-08-19

**Authors:** Lucie Vanikova, Eva Machackova, Barbora Nemcova, Jana Soukupova, Silvia Petrezselyova, Klara Novakova, Marcela Zenatova, Sarka Pavlova, Petra Kleiblova, Zdenek Kleibl, Lenka Foretova, Libor Macurek

**Affiliations:** 1 Cancer Cell Biology, Institute of Molecular Genetics of the Czech Academy of Sciences, 14220 Prague, Czech Republic; 2 Masaryk Memorial Cancer Institute, Brno, Czech Republic; 3 Institute of Medical Biochemistry and Laboratory Diagnostics, First Faculty of Medicine, Charles University and General University Hospital in Prague, Prague, Czech Republic; 4 Institute of Biology and Medical Genetics, First Faculty of Medicine, Charles University and General University Hospital in Prague, Prague, Czech Republic; 5 Department of Internal Medicine, Hematology and Oncology, and Institute of Medical Genetics and Genomics, University Hospital Brno and Medical Faculty, Masaryk University, Brno, Czech Republic; 6 Central European Institute of Technology (CEITEC), Masaryk University, Brno, Czech Republic; 7 Department of Pathophysiology, First Faculty of Medicine, Charles University, Prague, Czech Republic

**Keywords:** TP53, p53, Li Fraumeni syndrome, Cancer, Breast cancer, Tumour-suppressor proteins, Cancer genetics, Cancer prevention

## Abstract

Germline loss-of-function variants in *TP53* cause Li–Fraumeni syndrome (LFS) characterized by an early onset of various cancer types including sarcomas, adrenocortical carcinoma, and breast cancer. The most common are mutations in the DNA binding domain of p53, but alterations in the oligomerization domain also cause LFS with variable level of penetrance. Here we report identification of a novel germline in-frame deletion *TP53* variant c.1015_1023del p.(E339_F341del) in a family with early-onset breast cancer and other malignancies. Using functional testing, we found that a short deletion in the oligomerization domain in the p.E339_F341del variant severely impaired transcriptional activity of p53 in human cells and in a yeast model. The loss of the transactivation activity was consistent with an observed defect in formation of p53 tetramers. Finally, we found that cells expressing the p.E339_F341del variant were insensitive to inhibition of MDM2 by nutlin-3 confirming the functional defect. We conclude that the in-frame germline c.1015_1023del *TP53* variant encodes a transcriptionally inactive protein and promotes LFS with a high penetrant cancer phenotype.

**Supplementary Information:**

The online version contains supplementary material available at 10.1038/s41598-025-14684-8.

## Introduction

Tumor suppressor p53 acts as a transcription factor that triggers expression of various target genes involved in cell cycle, apoptosis, and metabolism. At basal conditions, level of p53 is low due to the constant ubiquitination by MDM2 and degradation by proteasome^1,2^. In response to various stress stimuli (including DNA damage, replication stress or oncogene activation), p53 is stabilized, forms a tetramer through its C-terminal oligomerization domain (OD; residues 325–356) and binds to the promoters of the target genes through its DNA binding domain (DBD; residues 102–292). Somatic mutations affecting the DBD are frequent in human tumors and typically associate with poor prognosis. In addition, germline *TP53* mutations cause Li–Fraumeni syndrome (LFS), a rare genetic disorder linked with early onset tumorigenesis including sarcomas, adrenocortical, breast and other cancer types^3^. Loss of function mutations are most common within the DBD and are less frequent in the OD^4,5^. Nevertheless, the oligomerization status of p53 has recently been linked with the level of penetrance in LFS families^6^. In addition, some of the mutants in the OD (in particular p.A347D) showed gain-of-function phenotypes that were unrelated to the transactivation activity^7,8^.

Attempts to identify the mechanism of tumorigenesis caused by the loss of p53 have failed to identify a single pathway whose defect could explain the cellular transformation in various tumor types, suggesting that the spectrum of the relevant p53-dependent transcriptional targets is large and may differ across tissues and cellular contexts^9,10^. The best understood function of p53 includes its ability to arrest the cell cycle progression by transcriptional activation of *CDKN1A* gene leading to production of the p21 protein, an efficient inhibitor of cyclin-dependent kinases^11,12^. Upon extensive genotoxic stress, p53 can promote apoptosis by various mechanisms including transcription of *PUMA*, *NOXA,* and other targets^13,14^. On the other hand, the genotoxic stress does not always lead to cell death as p53 also triggers expression of its negative regulator MDM2 thus limiting the extent of the pathway activation^15^.

During routine germline genetic testing, we identified a novel germline in-frame deletion c.1015_1023del; p.(E339_F341del) in the OD of p53 in a breast cancer patient with a positive family cancer history. To explore the pathogenicity of the variant, we performed a functional analysis of the p53-p.E339_F341del protein expressed in human RPE-TP53-KO cells and using the functional analysis of separated alleles in yeast (FASAY)^16^. We found that the variant isoform failed to induce expression of p21 and MDM2 in human cells and produced a functionally impaired protein in yeast model. In addition, we found that the p.E339_F341del isoform did not form tetramers. We conclude that *TP53* c.1015_1023del variant is a transcriptionally inactive germline pathogenic variant, which is responsible for a high cancer risk in the affected family.

## Results

### Identification of TP53 c.1015_1023del variant and clinical characteristics of the carrier

**Fig. 1:**
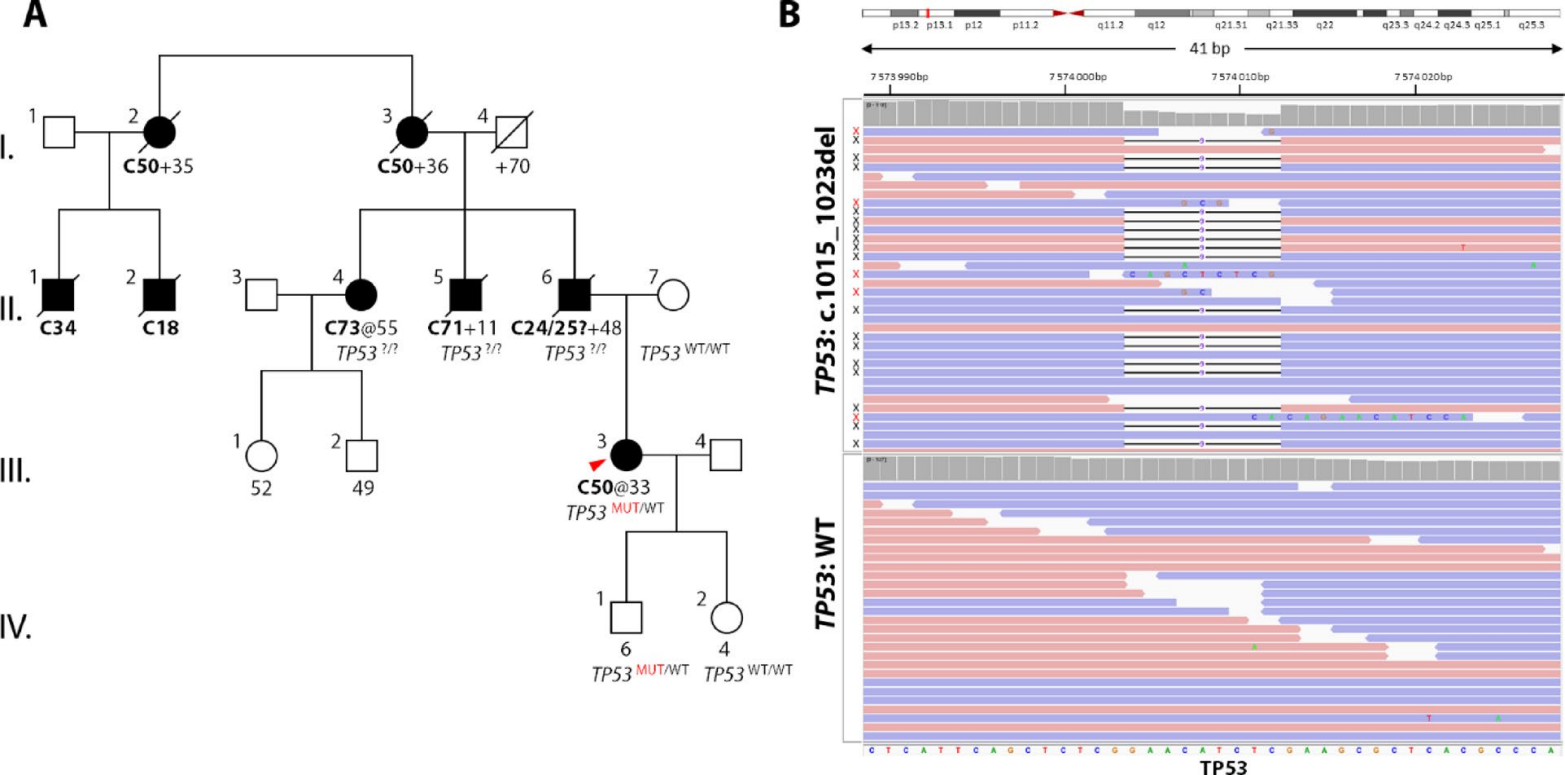
Identification of the p.E339_F341del variant. (**A**) The pedigree of the proband (red arrowhead) shows multiple cancer cases in the branch of the proband’s father (II/6), who died of biliary or pancreatic neoplasia at the age of 48. No detailed information was available for the offspring of the father’s aunt (I/2). C18 colon cancer; C34 lung cancer; C50 breast cancer; C24 biliary cancer; C25 pancreatic cancer; C71 brain tumor; C73 thyroid cancer. (**B**) NGS analysis of the proband revealed a deletion of nine nucleotides (c.1015_1023del) in the coding sequence of exon 10 (upper panel compared to the wild-type *TP53* sequence in the lower panel). The wild-type: altered allele ratio was 54:51, taking into account altered reads with a deletion (N = 41; indicated by black X) and incorrectly mapped reads with the deletion at their ends (N = 10; indicated by red X).

The healthy proband (born 1989) was initially tested in 2016 for the presence of germline pathogenic variants in *BRCA1* and *BRCA2* because of her positive family history. Her father died of pancreatic or hepatobiliary cancer at age 48, her uncle died of a brain tumor of uncertain histology at age 11 and her aunt was treated at age 55 for thyroid cancer (Fig. 1A). The father’s mother and her sister died of breast cancer at age 35 and 36, respectively. The initial *BRCA1* and *BRCA2* testing was negative. She was enrolled in breast cancer screening by yearly ultrasound. At the age of 33 years, the proband developed a discharge from her left breast and was diagnosed with synchronous breast cancer bilaterally. Ultrasonography identified a breast tumor (12 × 7 mm) with calcifications. Biopsy and histopathological examination showed an estrogen receptor (ER)-positive (90%), progesterone receptor (PR)-positive (90%), HER2 receptor-negative (0%), and Ki67-positive (45%) G3 ductal carcinoma in situ (DCIS). MRI revealed another nodular lesion with microcalcifications in the same breast and a similar lesion in the contralateral breast examined as multicentric breast cancer cT2/N0/M0. The patient underwent total bilateral mastectomy (without breast reconstruction) followed by a standard adjuvant treatment (doxorubicin/cyclophosphamide) and maintenance therapy with paclitaxel and is currently in her first complete remission. Due to the positive family cancer history, young age at the time of breast cancer diagnosis, and presence of bilateral tumor, we indicated an extension of the germline genetic testing by NGS using the CZECANCA panel targeting 226 risk genes^17^. The analysis revealed a previously undescribed in-frame heterozygous deletion in the *TP53* gene (NM_000546.6): c.1015_1023del, p.(Glu339_Phe341del; further referred to as E339_F341del; Fig. 1B) located in exon 10 coding for the conserved tetramerization domain of the p53 protein. Cascade germline genetic testing revealed that her son carries the same *TP53* heterozygous variant, whereas her daughter and the healthy mother carries the wild type *TP53*.

### Functional analysis of the TP53 c.1015_1023del variant

**Fig. 2:**
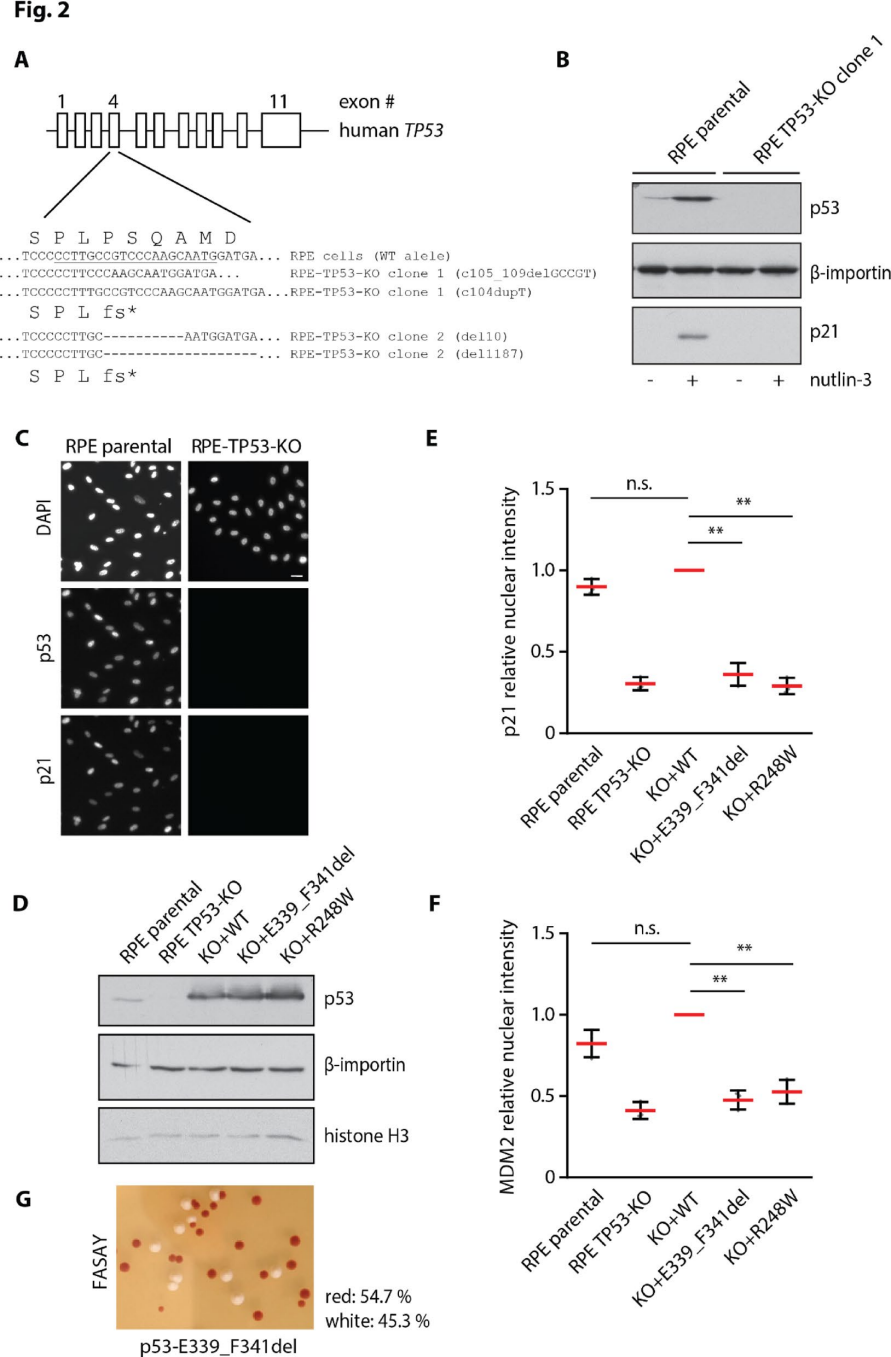
Impaired transcriptional activity of the p53 p.E339_F341del isoform. (**A**) Map of the *TP53* genetic locus targeted by CRISPR/Cas9. Parental RPE cells were transfected by sgRNA and Cas9, were grown in the presence of nutlin-3 and two clones of RPE-TP53-KO cells were expanded. Genomic DNA was sequenced by NGS. Partial sequence of exon 4 of the *TP53* is shown with the target sequence of sgRNA underlined. Note two frameshifting mutations corresponding to the two alleles in RPE-TP53-KO cells. (**B**) Whole cell lysates from parental RPE and RPE-TP53-KO cells incubated or not with nutlin-3 for 12 h were analyzed by immunoblotting. Note induction of p53 and p21 signal after nutlin-3 treatment in parental cells and the absence of p53 and p21 signal in RPE-TP53-KO cells. Staining for importin beta which is an abundant protein involved in nucleocytoplasmic trafficking was used as a loading control^19^. (**C**) Parental RPE and RPE-TP53-KO cells treated with nutlin-3 for 12 h were fixed by PFA, permeabilized by 0.1% TX-100 and analyzed by immunofluorescence microscopy. Representative image is shown. (**D**) Parental RPE, RPE-TP53-KO and RPE-TP53-KO cells stably transfected with wt-p53 (positive control), p53-R248W (negative control) and E339_F341del plasmids were treated with doxycycline and nutlin-3 for 12 h. Whole cell lysates were analyzed by immunoblotting. Staining for importin beta and histone H3 was used as loading controls. (**E**) Parental RPE, RPE-TP53-KO and RPE-TP53-KO cells stably transfected with wt-p53, p53-R248W and E339_F341del plasmids were treated with doxycycline and nutlin-3 for 12 h. After fixation and permeabilisation, cells were probed with p21 and p53 antibodies and analyzed by ScanR microscopy. Mean nuclear intensity of p21 signal was determined in > 300 non-gated RPE and RPE-TP53-KO cells or in the p53-positive RPE-TP53-KO cells rescued by the wild-type or mutant p53. Plotted is the mean ± SD from independent biological replicates (n = 3) normalized to p21 levels in cells expressing the wild type p53. Statistical significance was determined by t-test, **P < 0.01. (**F**) Parental RPE, RPE-TP53-KO and RPE-TP53-KO cells stably transfected with wt-p53 (positive control), p53-R248W (negative control) and E339_F341del plasmids were treated as in (**E**) and were probed with MDM2 and p53 antibodies. Mean nuclear intensity of MDM2 signal was determined as in (**E**). (**G**) FASAY analysis of the p53-E339_F341del variant transformed into yeast strain yIG397. White colonies (45.3%) contain the functional p53. The fraction of red colonies containing a transcriptionally inactive p53 allele was 54.7%, indicating that the patient is a heterozygote carrying one functional and one transcriptionally inactive p53 allele. Representative image is shown.

To perform a functional analysis of a newly identified *TP53* variant, we developed an experimental model of human non-transformed RPE1-TERT cells with frameshifting mutations in exon 4 of the *TP53* locus introduced using CRISPR/Cas9 technology (Fig. 2A). Using parental RPE1-TERT cells as controls, we confirmed the absence of p53 in RPE-TP53-KO cells by immunoblotting and by immunofluorescence microscopy (Fig. 2B,C). As expected, we also found that RPE-TP53-KO cells failed to induce expression of p21 (transcribed from the *CDKN1A* gene) after treatment with the MDM2 inhibitor nutlin-3 (Fig. 2B,C) or after exposure to ionizing radiation (data not shown). Next, we stably transfected RPE-TP53-KO cells with pCW57-RFP-P2A-TP53 plasmid allowing for doxycycline-inducible expression of the wild-type p53 (Fig. 2D). In parallel, we developed stable cell lines inducibly expressing the cancer-derived hot spot TP53-R248W mutant in the DNA-binding domain or the tested TP53-E339_F341del variant (Fig. 2D). Upon induction of the polyclonal cell lines with doxycycline, we observed a variable level of p53 expression in individual cells (data not shown) and therefore we used a high-content microscopy that allows gating for cells with comparable levels of p53 and we quantified the level of p21 expression. Importantly, we observed that induction of the wild-type p53 in RPE-TP53-KO cells stimulated comparable expression of p21 to that in the parental RPE cells treated with nutlin-3 (Fig. 2E). As expected, we found that the TP53-R248W mutant failed to promote expression of p21 (Fig. 2E). Interestingly, cells expressing the TP53-E339_F341del mutant also did not express p21 suggesting that this variant fails to transactivate the target genes of p53 (Fig. 2E). To exclude the possibility that the observed phenotype is specific for promoter of the *CDKN1A* gene, we repeated the analysis and quantified levels of MDM2 which is another transcriptional target of p53^18^. Indeed, we observed that the wild-type p53 stimulated expression of MDM2, whereas the TP53-R248W mutant failed to do so (Fig. 2F). Similarly, we found that the p53-E339_F341del mutant also did not stimulate expression of MDM2 thus fully confirming its impaired transcriptional activity (Fig. 2F). Finally, we tested the proband’s blood sample using an established FASAY assay in yeast. We observed 54.7% of red colonies representing the transcriptionally inactive p53 allele and 45.3% of white colonies representing the functional allele, which is consistent with an inactive TP53 variant present in the heterozygous state (Fig. 2G, Supplementary Fig. 1). Subsequent DNA sequencing of the red colonies confirmed the presence of the TP53-E339_F341del variant. Overall, the yeast assay fully confirmed the conclusion from the human cells that TP53-E339_F341del variant is transcriptionally inactive.

### Impaired tetramerization of the p53-E339_F341del protein

**Fig. 3:**
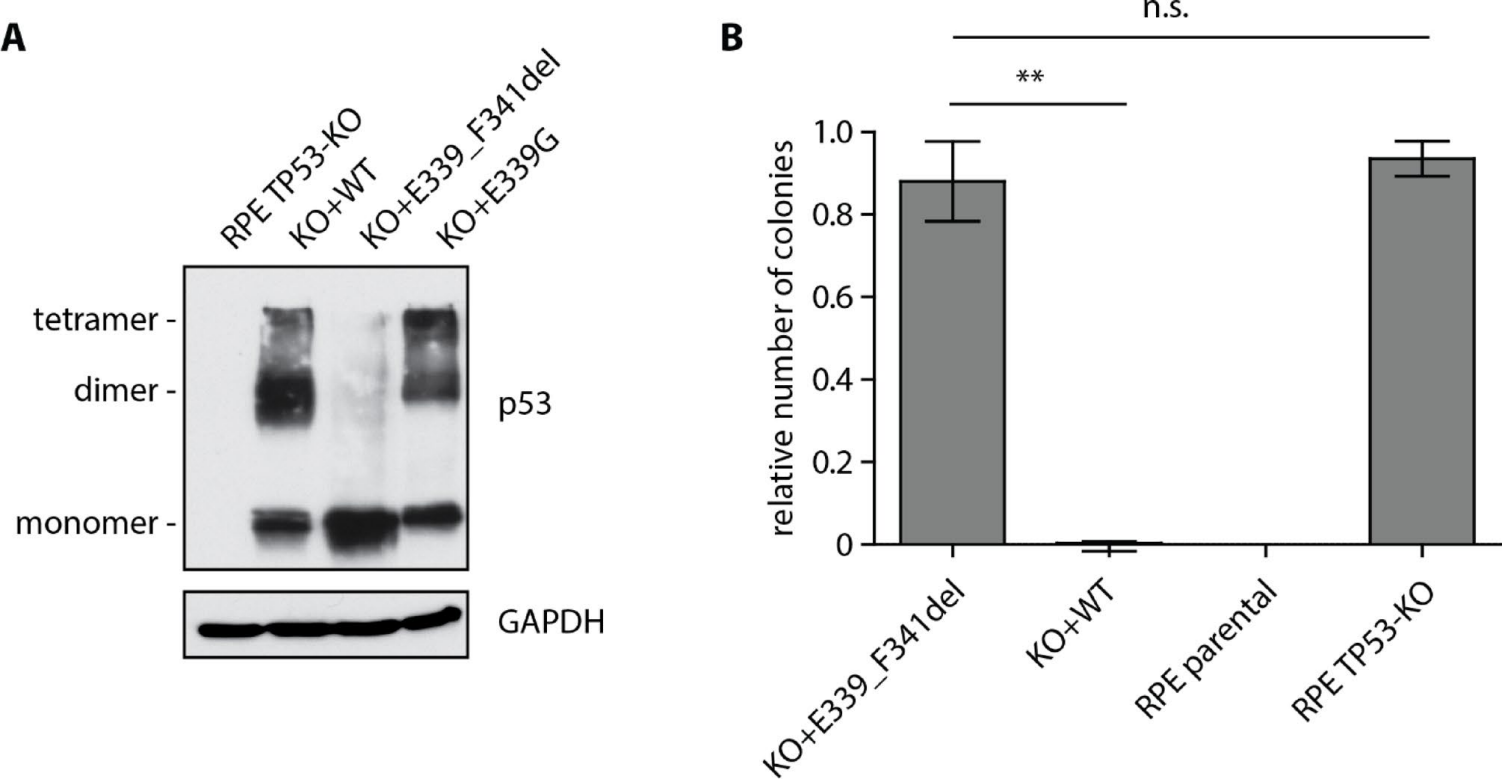
Impaired tetramerization of the TP53-E339_F341del mutant. (**A**) RPE-TP53-KO and RPE-TP53-KO cells stably transfected with wt-p53, p53-E339G and p53-E339_F341del plasmids were treated with doxycycline and nutlin-3 for 12 h. Cell extracts were cross-linked with glutaraldehyde and separated on SDS-PAGE. Staining for GAPDH protein was used as a loading control. Representative image from three experiments. (**B**) RPE-TP53-KO and RPE-TP53-KO cells stably transfected with wt-p53 (positive control) or p53-E339_F341del plasmids were treated with doxycycline, seeded on 6-well plates and were grown in the presence of nutlin-3 for 10 days. Upon fixation, the number of colonies was counted and normalized to the RPE-TP53-KO cells, error bars indicate SD, n = 3. Statistical significance was determined by t-test, **P < 0.01.

Mutations within OD with variable impact on p53 oligomerization have previously been reported in LFS^20,21^. In addition, the founder variant R337H that is linked with pediatric adrenocortical carcinoma in southern Brazil, has also been shown to exhibit decreased stability of the tetramer^22^. As the in-frame deletion p53-E339_F341del affects the alpha helix in the OD, we aimed to test its oligomerization properties. We treated cell extracts with 0.05% glutaraldehyde to cross-link the protein complexes and as expected, we observed that the wild type p53 formed dimers and tetramers (Fig. 3A). Similarly, another p53 variant E339G was able to form oligomers albeit with a slightly reduced efficiency compared to the wild-type p53 and this corresponded well to its preserved transcriptional activity (Fig. 3A, data not shown). In contrast, only the monomer was observed in E339_F341del mutant suggesting that its ability to form dimers and tetramers was strongly impaired (Fig. 3A).

Finally, we tested the impact of E339_F341del variant on cellular proliferation. As expected, we found that parental RPE cells failed to form colonies when cultivated in the presence of nutlin-3 whereas the RPE-TP53-KO cells were insensitive to inhibition of MDM2 (Fig. 3B). Expression of the wild type p53 efficiently suppressed proliferation of RPE cells in the presence of nutlin-3. In contrast, the expression of the E339_F341del mutant did not significantly affect the proliferation of cells thus confirming the loss-of-function phenotype (Fig. 3B).

Overall, we conclude that the impaired transcriptional activity of the newly identified the p53-E339_F341del variant is caused by its failure to form stable oligomers. Our complex functional analysis showed loss of function of the p53-E339_F341del variant in both the RPE human cells and in a yeast model. Applying our results within the strict framework of the ACMG/AMP Variant Interpretation Guidelines for *TP53* (v2.3.0) supports classification of the variant as a VUS, based on the following codes: PS3 (4 points), PS4 (0.5 point), PM2 (1 point)^23^. As GVGD and BayesDel tools are not suitable for in-frame deletions, we analyzed the c.1015_1023del *TP53* variant by alternative prediction algorithms that all supported a damaging effect, including MutPred-Indel (score: 0.82806; likely deleterious, high confidence), MutationTaster (deleterious), FATHMM-indel (score: 0.9392; pathogenic), and PROVEAN (score: − 16.22; deleterious). The concordant outcome of the predictions justified application of the additional PP3 criterion (1 point) and conclusion that the c.1015_1023del variant could be considered likely pathogenic*.* The variant classification is consistent with the clinical data suggesting association of the *TP53*-E339_F341del variant with the development of early onset tumors typical for LFS.

## Discussion

The introduction of NGS into clinical genetic testing for germline cancer predisposition has rapidly increased both the number of patients analyzed and the number of germline variants identified. For several reasons, the female breast cancer patients are analyzed the most frequently. Breast cancer is the most common in female cancer patients worldwide, the population frequencies of pathogenic variants in breast cancer predisposition genes belongs to the highest among the high-penetrant cancer predisposition genes overall (especially in *BRCA2* and *BRCA1*), the risk of cancer in carriers of pathogenic variants is well described and the guidelines for their cancer prevention and treatment are available in the clinical practice worldwide^24–26^. The *TP53* gene belongs to routinely tested breast cancer predisposition genes, regardless of the rarity of its germline variants, which are two orders of magnitude less frequent than those in *BRCA2* (0.06% vs. 1.29% and 0.014% vs. 1.54%, respectively), as demonstrated in the largest breast cancer studies by Hu et al. and the Breast Cancer Association Consortium^27,28^. The evidence that breast cancer, and in particular its early-onset form, is the most common type of tumor in female patients with LFS provides a rationale for the germline analysis of *TP53*. Breast cancer was observed in 79% of female LFS patients in a French population with median age at disease onset at 33 years^29^. The German LFS Registry study found breast cancer in over 35% of LFS cancer patients (15% at the age of ≤ 30 years and 20% at age > 30 years) and in > 50% of all cancers in patients with attenuated LFS^30^. The indication for germline genetic testing of *TP53* is recommended in all women diagnosed with breast cancer up to 31 years of age. The carriers of *TP53* pathogenic variants were found in 6% of such patients in aforementioned French study and similar frequencies (3.8–7.7%) were reported by Fortuno et al. who reviewed 59 breast cancer studies analyzing the germline *TP53* variants in female patients diagnosed at age < 30 years^29,31^. Importantly, about half of the *TP53* carriers did not meet the NCCN criteria for LFS just like our proband developing HER2-negative (different from pathognomonic HER2-positive tumors in early-onset LFS patients) at the age of 33 years^32^. Interestingly, a reduced proportion of HER2-positive tumors has been found also in patients carrying the p.R337H variant, Brazilian founder variant and the most frequent pathogenic alteration affecting the OD in p53.

The clinical significance of LFS patients and a high frequency of somatic *TP53* variants have stimulated functional studies aimed at classifying *TP53* variants^33^. The largest analyses have implemented saturation editing approaches, which allow comprehensive analyzes of variants across the entire coding sequence^34,35^. However, these approaches, recommended for the application of the PS3 criterion for ACMG/AMP *TP53*-specific variant interpretation guidelines do not consider in-frame variants including c.1015_1023del found in our proband^23^. Since there were no functional data available for the variant, we have tested the pathogenicity of the variant using functional assays in human cells and in yeast. In agreement with the reported correlation between the oligomerization status and the clinical outcome of LFS^6^, we observed a high penetrance of various types of cancer in the family carrying the germline *TP53* c.1015_1023del variant that results in production of oligomerization-deficient E339_F341del protein. Our functional analysis in human RPE cells revealed that E339_F341del fails to stimulate the expression of two independent p53 targets suggesting that its pathogenic behavior is likely caused by transactivation defect in transcription. In contrast, we tested the p.E339G missense variant that was able to form oligomers and showed no defects in transcription of p21 and MDM2, which is in good agreement with a previous description of the p.E339G variant as benign^36^. The pathogenic nature of the p.E339_F341del variant was further supported by the FASAY showing impaired transactivation of the p53 promoter in a yeast experimental system. Our results are consistent with previously published data by Kawaguchi et al., who conducted a systematic mutational analysis of individual amino acids in the OD^37^. The authors demonstrated that substitutions at F340 and F341, but no at E339 (all comprising part of the α-helix critical for tetramer formation) impaired oligomerization and severely reduced transcriptional activity. Loss of the tetrameric structure is directly linked to the loss of p53 function, and deletions encompassing this segment may have an even greater destabilizing effect than individual missense variants.

Although our functional analyses showed that the c.1015_1023del variant encodes functionally impaired p53 protein with in frame deletion of tree amino acids in the OD, the lack of samples from deceased relatives of the proband hindered the segregation analysis. We found no other carrier of this variant in the analysis of over 50,000 Czech probands tested by panel NGS suggesting that the variant is probably rare. On the other hand, it has been reported in the COSMIC database (COSM23457149) and also as a somatic variant in a non-small cell lung cancer tumor sample with LOH in the second allele^38^.

In conclusion, we provide evidence that the in-frame germline c.1015_1023del *TP53* variant encodes a transcriptionally inactive p53-E339_F341del and promotes LFS with early onset of various types of cancer.

## Methods

### Probands

The proband fulfilling the hereditary breast cancer germline genetic testing criteria was initially tested for germline variants in *BRCA1* and *BRCA2* in 2015 and then re-analyzed in 2022 using the CZECANCA panel NGS^17,39^. None of the relatives with cancer were available for genetic testing. All healthy relatives who consented to genetic testing underwent targeted, variant-specific germline genetic testing. All participants provided informed consent during the study. The study was approved by the Ethical committee of the Masaryk Memorial Cancer Institute (OEGN/1) and was performed in accordance with the Declaration of Helsinki.

### Antibodies and reagents

The following antibodies were used: p53 (sc-6243, IF dilution 1:100), p21 (sc-6246, IF dilution 1:100, WB 1:1000), and importin beta (sc-137016) from Santa Cruz; MDM2 (OP46, IF dilution 1:100) from Calbiochem; histone H3 (14269S, WB 1:1000), GAPDH (5174S, WB 1:1000) and p53 (9282S, WB 1:1000) from Cell Signaling Technology; Alexa Fluor-conjugated secondary antibodies (Thermo Scientific). MDM2 inhibitor nutlin-3 (Medchemexpress) was diluted in DMSO and used at final concentration 9 µM^40^.

### SDS-PAGE and immunoblotting

Protein electrophoresis of the whole cell lysates (20 µg) was performed as described previously using 5–20% gradient gels and Tris/Glycine/SDS solutions (Bio Rad)^41–43^. After western blotting, nitrocellulose membrane was probed with the indicated primary and HRP-conjugated secondary antibodies and the signal was developed using ECL reagents and X-ray films. Tested proteins (p53 or p21) and the loading controls (histone H3, GAPDH or importin beta) were analyzed from the identical gel exploiting their different electrophoretic mobility.

### Plasmids

DNA fragment carrying the coding sequence of human *TP53* was inserted into pCW57-RFP-P2A-MCS plasmid (Addgene ID: 78933) allowing for doxycycline-inducible expression of turbo RFP reporter and p53. Subsequently, c.1015_1023del variant coding for p53-E339_F341del was introduced into the plasmid by site directed mutagenesis. All plasmids were confirmed by Sanger sequencing.

### Cells

Immortalized human retinal pigment epithelia RPE1-hTERT cells (referred to as RPE) were from ATCC. Cells were grown in high-glucose DMEM supplemented with 6% FBS (Gibco), Penicillin (10 U/mL) and Streptomycin (0.1 mg/mL). RPE-TP53-KO cells were generated by transfection of RPE cells by a complex of sgRNA CAUUGCUUGGGACGGCAAGG (Sigma) targeting exon 4 of the *TP53* gene and purified TrueCut protein Cas9 v2 using CRISPRMAX (Thermo Fisher Scientific). Selection of cells with inactivated *TP53* was performed by cultivation in the presence of nutlin-3 for 3 weeks followed by expansion of single cell clones. Complete loss of p53 expression was confirmed by immunoblotting and by immunofluorescence microscopy. Stable cell lines were generated by transfection of RPE-TP53-KO cells with pCW57-RFP-P2A-TP53-WT or c.1015_1023del plasmids followed by selection with geneticin for 3 weeks and flow cytometry-mediated sorting of RFP-positive cell after short (6 h) pulse of doxycycline.

### Quantitative microscopy

Parental RPE and RPE-TP53-KO cells stably transfected with pCW57-RFP-P2A-TP53-WT or pCW57-RFP-P2A-TP53-c1015_1023del plasmids were treated with doxycycline (2 µg/mL) and nutlin-3 (9 µM) overnight. Cells grown on coverslips were fixed by 4% PFA, permeabilized with 0.1 TX-100 in PBS and incubated with p53 and MDM2 or p53 and p21 antibodies for 2 h. After washing with PBS, cells were incubated with Alexa conjugated secondary antibodies for 2 h and finally, after washing with PBS coverslips were mounted with Vectashield containing DAPI. Imaging was performed using Olympus ScanR station equipped with UPLXAPO 40×/0.95 DRY CORR objectives. Image analysis was performed by Olympus ScanR analysis v3.2 (https://evidentscientific.com/en/products/high-content-screening/scanr) and FlowJo v9 software (https://flowjo.com/flowjo/) was used for data visualization. Cells expressing comparable levels of p53 as the parental RPE cells were gated and median nuclear intensity of MDM2 or p21 signal was determined. Median value was determined from at least 300 individual cells per condition.

### Statistics

All experiments were performed in three or more biological repeats. Data visualization and statistical analysis was performed using Prism 5 (GraphPad Software, https://www.graphpad.com/). Plots show means, error bars show standard deviations. Unpaired two-tailed T-test was used for evaluation of statistical significance, ****P < 0.0001, ***P < 0.001, **P < 0.01, *P < 0.05.

### Oligomerization assay

The ability of p53 mutants to form tetramers was evaluated using a previously reported protocol^44^. Expression of p53 or p53-E339_F341del mutant was induced in reconstituted RPE-TP53-KO cells by doxycycline. Cells were incubated with lysis buffer (10 mM Tris–HCl, pH 7.5, 1 mM EDTA, 1% Triton X-100, 150 mM NaCl, 1 mM dithiothreitol, 10% glycerol, 0.2 mM phenylmethylsulfonyl fluoride, and protease inhibitors) for 30 min on ice, spinned down and 0.05% glutaraldehyde was added to the extracts. After 15 min, extracts were mixed 1:1 with the 2 × Laemli buffer and analyzed by immunoblotting using polyclonal p53 antibody.

### Colony formation assay

One thousand of parental RPE, RPE-TP53-KO or freshly sorted RFP-positive RPE-TP53-KO cells reconstituted with the wild-type p53 or p53-E339_F341del mutant were seeded into 6-well-plates and were grown in the presence of doxycycline and nutlin-3. After 10 days, cell colonies were washed with PBS, fixed and stained with 1% crystal violet in 20% ethanol for 30 min. The colonies were counted manually using ImageJ.

### FASAY analysis

FASAY analysis was performed as described previously^16,45^. Briefly, RNA isolated from peripheral blood was transcribed to cDNA using Superscript II Reverse Transcriptase (Thermo Scientific) and oligo dT. Fragment corresponding to codons 42–374 of the *TP53* was PCR amplified and was transformed together with the linearized pSS16 vector into yeast strain yIG397 containing ADE2 open reading frame controlled by a p53 responsive promoter. Yeast were cultivated on a selection plate without leucine and with minimal amount of adenine at 35 °C and the fraction of red colonies containing the transcriptionally inactive p53 was calculated. DNA was sequenced from four red colonies and confirmed the presence of the tested *TP53* variant.

### Prediction software tools

The following prediction software tools were used for evaluation of the c.1015_1023del *TP53* variant: BayesDel https://fenglab.chpc.utah.edu/BayesDel/BayesDel.html, GVGD (http://agvgd.hci.utah.edu/about.php), MutationTaster (https://www.mutationtaster.org/), MutPred-Indel (http://mutpred2.mutdb.org/mutpredindel/about.html)^46^, FATHMM (http://indels.biocompute.org.uk/)^47^, and PROVEAN (https://www.jcvi.org/research/provean).

## Supplementary Information

Below is the link to the electronic supplementary material.

Supplementary Material 1

Supplementary Material 2

## Data Availability

All data generated during this study are included in this published article and its Supplementary information file.
