# Supplementary material for: In-frame germline TP53 variant impairs p53 oligomerization and predisposes to cancer
Source: Sci Rep. 2025 Aug 19;15:30459. doi: 10.1038/s41598-025-14684-8 (PMC12365031; doi:10.1038/s41598-025-14684-8)
Supplement: Supplementary file 1 — Supplementary Material 1 [file 41598_2025_14684_MOESM1_ESM.pdf]

Figure 2B

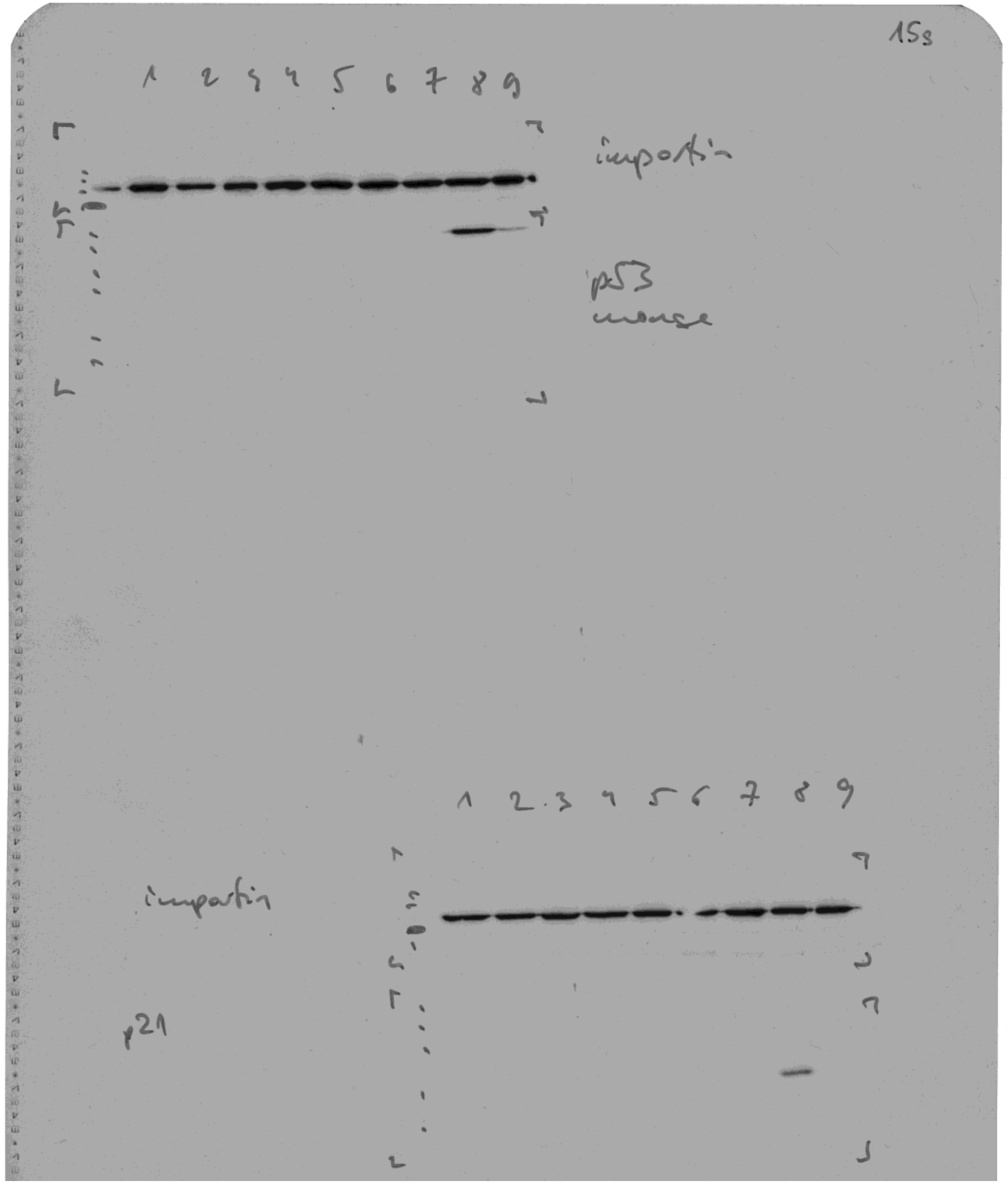

Figure 2D

Short exposure

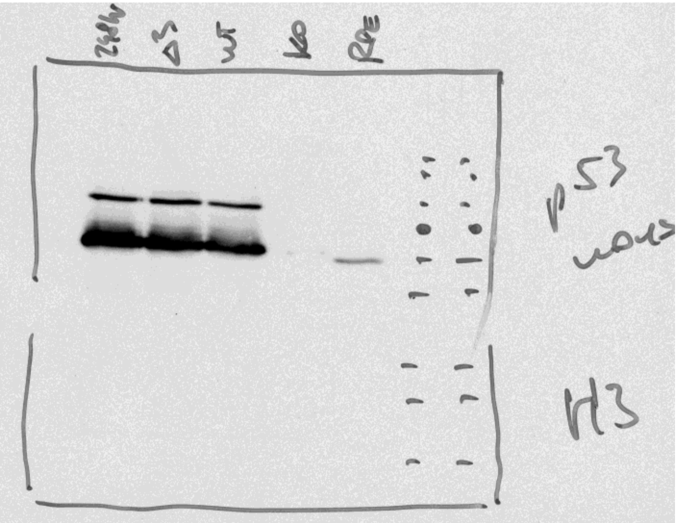

Long exposure

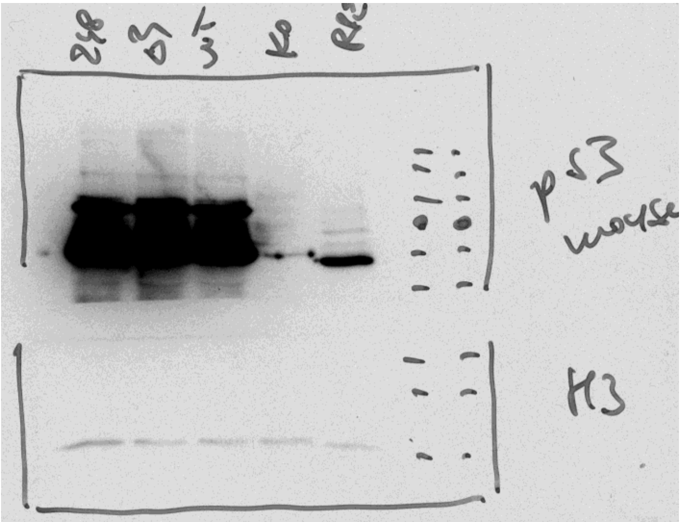

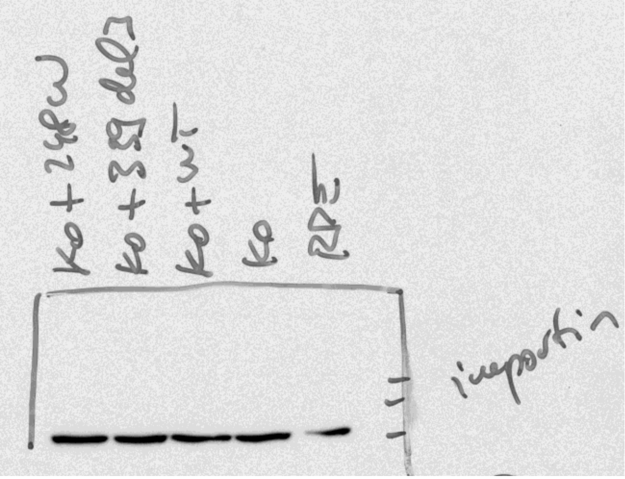

Figure 2G

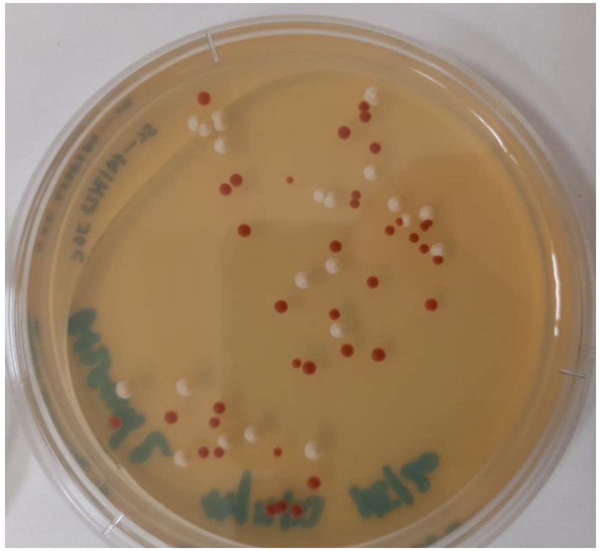

Figure 3A

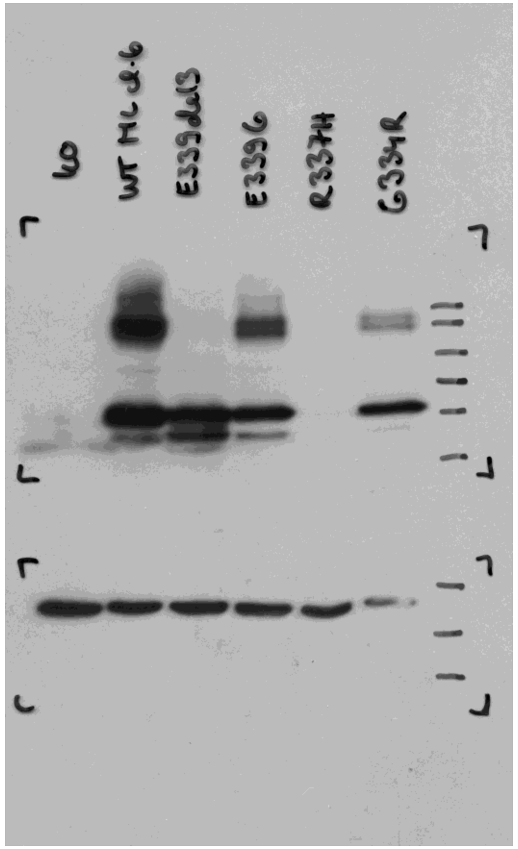

Figure 2E

	R248W	E339_F341del	WT	RPE parental	RPE TP53-KO
Mean nuclear intensity of p21 signal	0.346583	0.422049	1	0.88252	0.349844
	0.25182	0.283831	1	0.860581	0.276385
	0.270145	0.376324	1	0.953541	0.283943

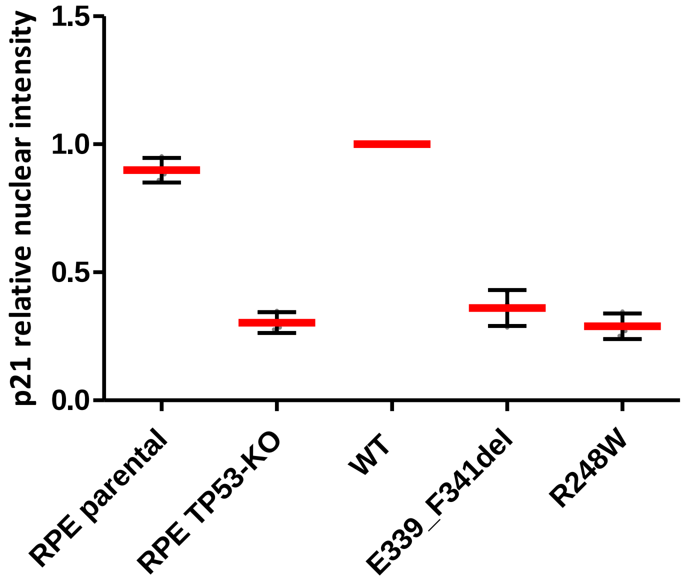

Figure 2F

	R248W	E339_F341del	WT	RPE parental	RPE TP53-KO
Mean nuclear intensity of MDM2 signal	0.607769	0.409437	1	0.728238	0.378465
	0.464467	0.515984	1	0.886452	0.385221
	0.508242	0.502355	1	0.854321	0.4722704

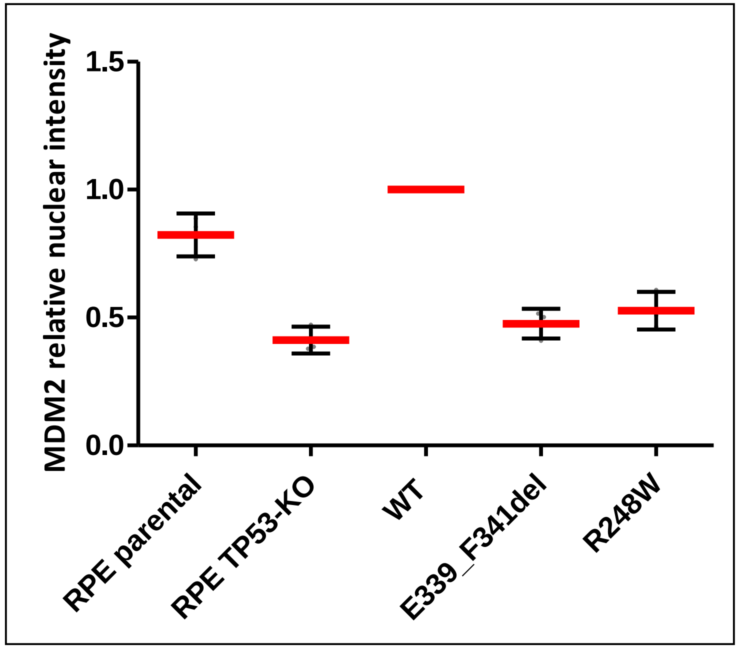

Data exported from PRISM software
