# Supplementary material for: In-frame germline TP53 variant impairs p53 oligomerization and predisposes to cancer
Source: Sci Rep. 2025 Aug 19;15:30459. doi: 10.1038/s41598-025-14684-8 (PMC12365031; doi:10.1038/s41598-025-14684-8)
Supplement: Supplementary file 2 — Supplementary Material 2 [file 41598_2025_14684_MOESM2_ESM.pdf]

Supplementary Figure 1

A

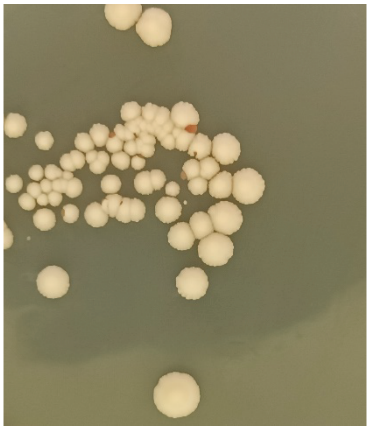

B

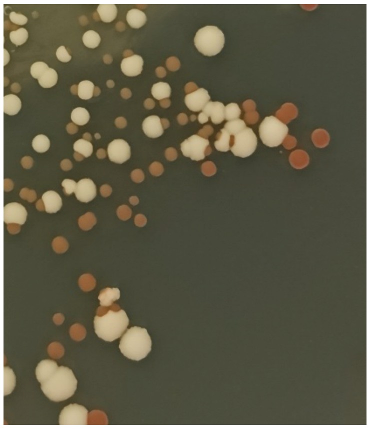

A: An example of FASAY result of a sample with a wild-type TP53. White colonies contain transcriptionally active, wild-type p53. Background of the red colonies (6.1%) represents polymerase-induced errors.
B: FASAY result of a sample with the heterozygous hot-spot variant p.R248Q (57.3% of red colonies with transcriptionally inactive p53).
